# An Overview of Nanoparticle Protein Corona Literature

**DOI:** 10.1002/smll.202301838

**Published:** 2023-04-29

**Authors:** Mohammad J. Hajipour, Reihaneh Safavi-Sohi, Shahriar Sharifi, Nouf Mahmoud, Ali Akbar Ashkarran, Elizabeth Voke, Vahid Serpooshan, Milad Ramezankhani, Abbas S. Milani, Markita P. Landry, Morteza Mahmoudi

**Affiliations:** Department of Radiology and Precision Health Program, Michigan State University, East Lansing, MI 48824, USA; Department of Radiology, Stanford University, Stanford, CA 94304, USA; Department of Radiology and Precision Health Program, Michigan State University, East Lansing, MI 48824, USA; Department of Chemistry and Biochemistry, University of Notre Dame, Notre Dame, IN 46556, USA; Department of Radiology and Precision Health Program, Michigan State University, East Lansing, MI 48824, USA; Department of Radiology and Precision Health Program, Michigan State University, East Lansing, MI 48824, USA; Faculty of Pharmacy, Al-Zaytoonah University of Jordan, Airport Rd., 11733 Amman, Jordan; Department of Biomedical Sciences, College of Health, Sciences, QU Health, Qatar University, Doha 2713, Qatar; Department of Radiology and Precision Health Program, Michigan State University, East Lansing, MI 48824, USA; Department of Chemical and Biomolecular Engineering, University of California, Berkeley, CA 94720, USA; Wallace H. Coulter Department of Biomedical Engineering, Emory University School of Medicine and Georgia Institute of Technology, Atlanta, GA 30322, USA; Department of Pediatrics Emory University, Atlanta, GA 30322, USA; Children’s Healthcare of Atlanta, Atlanta, GA 30322, USA; School of Engineering University of British Columbia, Kelowna, BC V1V 1V7, Canada; School of Engineering University of British Columbia, Kelowna, BC V1V 1V7, Canada; Department of Chemical and Biomolecular Engineering, University of California, Berkeley, CA 94720, USA; Innovative Genomics Institute, Berkeley, CA 94720, USA; California Institute for Quantitative Biosciences, University of California Berkeley, Berkeley, CA 94720, USA; Chan Zuckerberg Biohub, San Francisco, CA 94158, USA; Department of Radiology and Precision Health Program, Michigan State University, East Lansing, MI 48824, USA

**Keywords:** analytical chemistry, characterization, nanomedicine, protein corona, reproducibility, standard procedures

## Abstract

The protein corona forms spontaneously on nanoparticle surfaces when nanomaterials are introduced into any biological system/fluid. Reliable characterization of the protein corona is, therefore, a vital step in the development of safe and efficient diagnostic and therapeutic nanomedicine products. 2134 published manuscripts on the protein corona are reviewed and a down-selection of 470 papers spanning 2000–2021, comprising 1702 nanoparticle (NP) systems is analyzed. This analysis reveals: i) most corona studies have been conducted on metal and metal oxide nanoparticles; ii) despite their overwhelming presence in clinical practice, lipid-based NPs are underrepresented in protein corona research, iii) studies use new methods to improve reliability and reproducibility in protein corona research; iv) studies use more specific protein sources toward personalized medicine; and v) careful characterization of nanoparticles after corona formation is imperative to minimize the role of aggregation and protein contamination on corona outcomes. As nanoparticles used in biomedicine become increasingly prevalent and biochemically complex, the field of protein corona research will need to focus on developing analytical approaches and characterization techniques appropriate for each unique nanoparticle formulation. Achieving such characterization of the nano-bio interface of nanobiotechnologies will enable more seamless development and safe implementation of nanoparticles in medicine.

## 1. Introduction

In recent years, nanotechnology has been touted as a major breakthrough for the detection and treatment of diseases.^[1]^ In nano-biomedical research, nanomaterials have been used as a means for drug delivery and diagnosing diseases.^[2]^ To improve the safety and therapeutic efficacy of nanomedicine technologies, it is imperative to gain a better understanding of how nanoparticles (NPs) interact with their biological environments.^[3,4]^ With the myriad of published studies, we now understand that the biomolecular corona (which consists of various types of biomolecules but mostly proteins) forms spontaneously upon introduction of NPs in biological tissues or fluids, and that the type, amount, and conformation of the proteins adsorbed on the NP surface are key factors regulating biocompatibility and, ultimately, the fate of the nanomaterial both in vitro and in vivo.^[5-9]^ The wealth of data published on the interaction of the NPs with biological media, especially proteins, provides a fundamental picture of NP-biological system interactions^[10,11]^ affecting biocompatibility and safety. Nanomaterial scientists are currently using a variety of methodological approaches to evaluate toxicity, interactions with biological media, and efficacy of commercial NPs or the NPs they synthesize in their own labs. A wide range of experimental conditions such as exposure times, concentrations, cell types, and media are used. Biological responses such as cytotoxicity or protein NP interactions are also measured using various analytical techniques. Hence, a comprehensive understanding of the interaction of NPs with biological systems is critical in designing and developing safe and efficient clinical diagnostic and therapeutic applications with approaches reflective of the vast physiochemical complexity of our nanoparticle-based therapeutics.^[3,4,12-14]^

While most efforts continue to explore new nanomaterial-based technologies, some efforts have begun to decipher the reasons behind the slow translation of nanopharmaceuticals. To improve the reproducibility of nanomedicine reports, Caruso and co-workers stress the need for standardization within the field of nanotoxicology, as results in the literature are hard to compare because of lack of consistency, poorly characterized nanomaterials, and non-standardized study conditions.^[15]^ These authors suggest that nanomedicine researchers use the checklist called Minimum Information Reporting in Bio-Nano Experimental Literature (MIRIBEL) to improve reproducibility. Although that idea found some support among researchers, the later reaction to the MIRIBEL showed that the requirements of physical, chemical, and biological characterization reporting of nanophar-maceuticals are much more complex than can be addressed by a checklist. Such checklist provide preproducibility (i.e., having enough information to repeat the experiments)^[16]^ for others to improve the reproducibility in their outcomes. In fact, a systematic approach and universally-relevant standards are needed to assure the safety of nanomedicine and, more importantly, to ensure that nano medicinal products and their raw materials properly characterized in the biofluids for their intended application.^[13,17]^

### 1.1. Protein Corona Analysis

**Figure 1.**
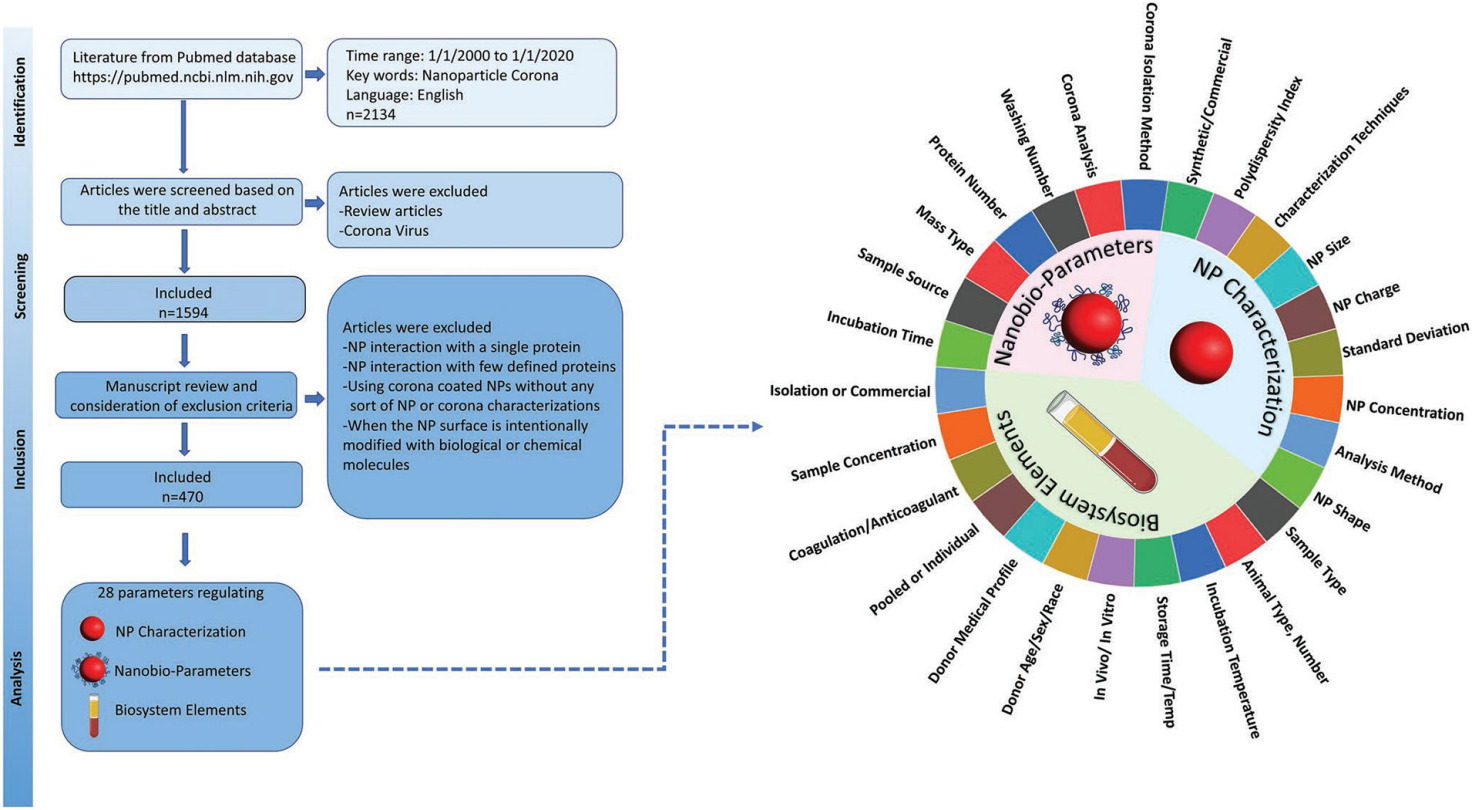
The methodology, screening, inclusion, and exclusion criteria for the literature search on protein corona formation on NPs. Searching the PubMed database with keywords “nanoparticle” and “corona” within 20 years yielded 2134 manuscripts, which were included in the initial database. Review manuscripts and papers related to the coronavirus were excluded, resulting in 1594 papers. Papers investigating protein corona formation with a single protein or just a few pre-determined proteins were also excluded, as were papers lacking physical, chemical or biological characterization of the formed protein corona around nanoparticles. A final 470 research manuscripts involving 1702 different NPs were identified and analyzed by application of three main analysis criteria: NPs characterization, nano-bio parameters, and biosystem elements (28 parameters).

The protein corona is a relevant phenomenon to investigate as i) its importance to NP safety and effectiveness has been well-established, and ii) obtaining, analyzing, and studying the protein corona has multiple verifiable steps, which allows us to investigate the reproducibility and quality of the data reporting in the published literature. We have surveyed the literature on the protein corona that forms on nanomaterial surfaces, and selected papers that meet criteria for deep analysis according to the strategies presented in Figure 1, excluded reports i) investigating protein corona formation with a single protein or few defined proteins, ii) that are unclear about the procedures employed in protein corona formation, iii) that lack physical, chemical, or biological characterization of the formed protein corona. We analyzed a total of 1702 NPs extracted from 470 manuscripts, including 28 parameters within three classes of NP characteristics: nano-bio parameters, NP characterization, and biosystem elements (see Figure 1 and Table S1, Supporting Information). All of these parameters are critical in driving corona formation or its analysis. For example, NP size, charge, concentration, characterization technique, etc., are critical in determining the protein corona, and as such these values and the details of their characterization need to be reported to allow proper comparisons between studies. Similarly, in terms of biosystem elements, the type and concentrations of biological media, exposure time, storage temperature, etc., all affect protein corona formation, and those experimental details need to be reported. Likewise, details of corona analysis and preparation such as number of washes and incubation time can also affect protein corona formation and need to be reported in detail to permit reproducibility.

**Figure 2.**
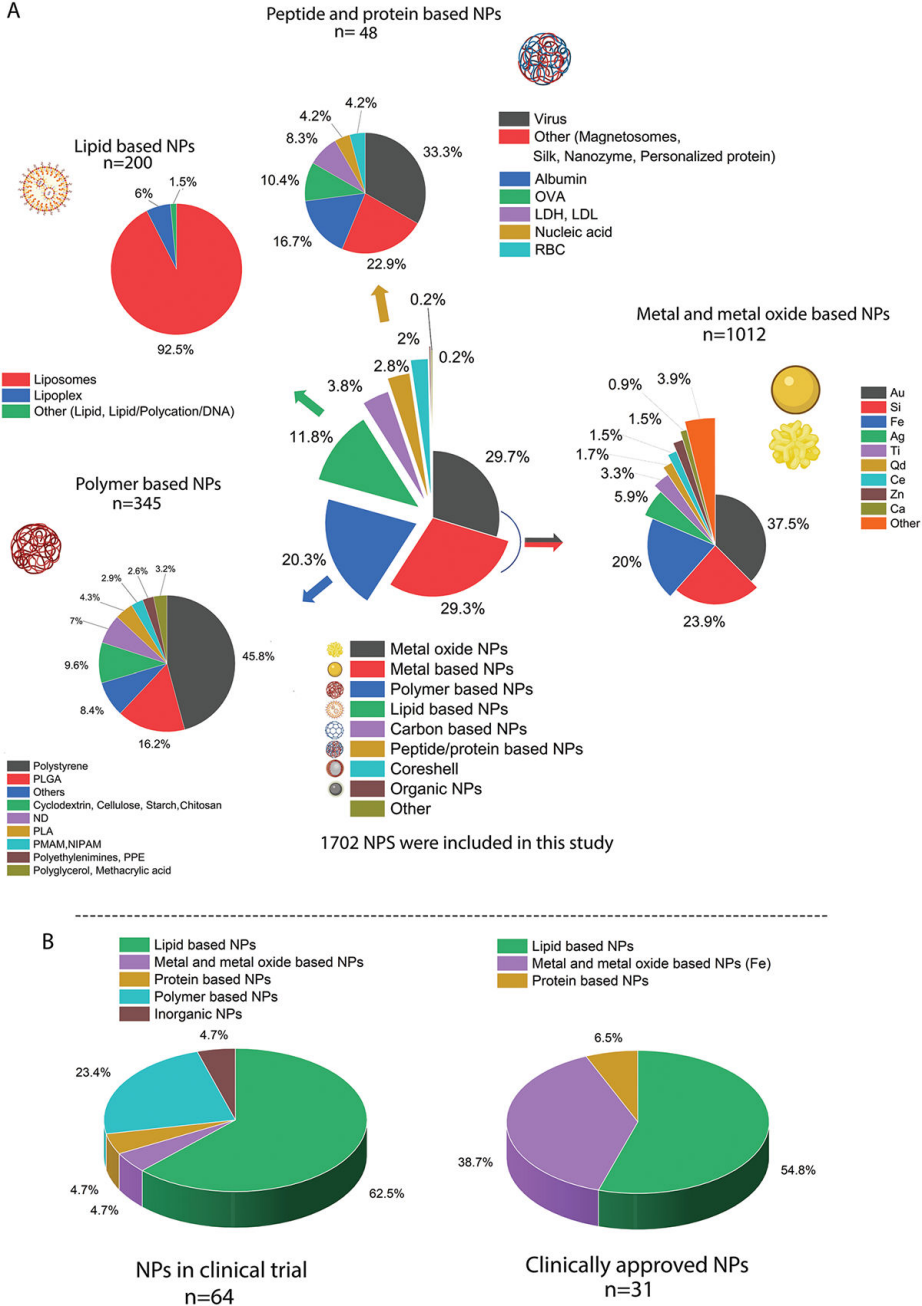
A) A detailed representation of the composition of all NPs directly or indirectly used in studying the formation and evolution of protein corona around NPs. B) clinically approved NPs therapies, diagnostics, and intravenous NP clinical trials that are currently undergoing clinical trials (not yet recruiting, recruiting, or active). The data for figures were extracted and reconstructed into composition groups from ref. [25]. In both groups, lipid based and specially liposomes are the dominantly used NPs in the clinic.

A detailed outline of the NP compositions used in the surveyed protein corona research is presented in Figure 2A. We divided NP compositions into seven main groups: metal-based, metal oxide-based, polymer-based, lipid-based, carbon-based, core-shell or composite NPs, and other. For simplicity, we include studies on silicon-based NPs in the metal and metal oxide groups. Separately, we also analyzed the composition of NPs currently undergoing clinical trials or approved for clinical use, including intravenously administered NPs (Figure 2B). Interestingly, the majority of the NPs on the market or undergoing clinical trials are lipid-based (mainly liposomes), followed by iron-based NPs for anemia treatment. Notably, despite their overwhelming presence in clinical practice, lipid-based NPs are vastly underrepresented in protein corona research.

A significant portion of nanoparticle protein corona information in the literature (59% of all studied NPs) has been gathered using metal-based and metal oxide–based NPs, with those incorporating gold, silicon, and iron being the most abundant (380 NPs out of 1013 NPs are gold). Interestingly, despite extensive corona research on gold NPs, no NP gold-based treatment has reached the clinic.^[18]^ Similarly, despite the plethora research on silicon NP toxicity, substantial obstacles such as safety from short-term exposure and long-term toxicological profiles need to be addressed before moving silicon NPs into the clinic.^[19]^ Moreover, more reliable scale-up methods and minimizing batch-batch variation in silica NPs remain to be resolved for use in humans.^[19]^ The success of these ongoing endeavors is highly dependent on proper characterization of nanomaterials and their interactions with biological systems, combined with an efficient reporting system that ensures reproducibility and reliability of protein corona data.

Polymeric NPs—with polystyrene NPs in the lead (158 NPs out of 345 polymeric NPs)—account for 20.3% of the NPs studied here. Polystyrene NPs are hard NPs and therefore are easier to work with than polymeric NPs that are soft and therefore more easily perturbed in the experimental workflows necessary to recover and isolate the protein corona from the NP surface. Other soft polymeric NPs are the second largest group of materials used for therapeutic purposes, including cancer treatment in several clinical trials (Figure 2B). Lipid-based NPs were used in 11.8% of protein corona studies. Protein and peptide-based NPs were among the least used NPs in our data set (2.8% of all studies), and virus-based particles were the leaders in the protein and peptide groups, followed by albumin and then ovalbumin. Generally, we consider hard NPs, such as polystyrene NPs or gold NPs, to be those that do not dissociate under the perturbative conditions used to isolate the protein corona (buffer exchanges, high speed centrifugation) and soft NPs, such as liposome or lipid-based NPs, to be those who could change in their structural or chemical identity under those conditions.

Among all types of NPs, those based on lipids are the most established and are widely used in clinical use, including the delivery of molecules such as anticancer drugs, mRNA therapeutics,^[20]^ or imaging or treating age-related diseases such as macular degeneration. The first FDA-approved nanomedicine was liposomal doxorubicin (Doxil) which was approved in 1995.^[21]^ More than half a century of history, Kinsky et al.^[22]^ investigated the mechanism of blood complement protein activation in the presence of liposomes and complement-mediated membrane damage. Initial studies on protein material interactions (often referred to as opsonization) were focused mainly on the interaction of opsonin proteins with liposomes, which plays a critical role in their stability and clearance from the blood circulation.^[23]^ The term “protein corona” however was not coined until 2007.^[24]^ Unlike the earliest protein material interaction studies, recent protein corona studies are focused on using mass-spectrometry-based proteomics to identify the large variety of the proteins bound to the NP surface. Based on our analysis, the majority of NPs corona research is focused on hard NPs (metals, metal oxides and polymer NPs) which covers 79% of all studies. Soft NPs such as proteins or lipid based are about 14% of all studies.

**Figure 3.**
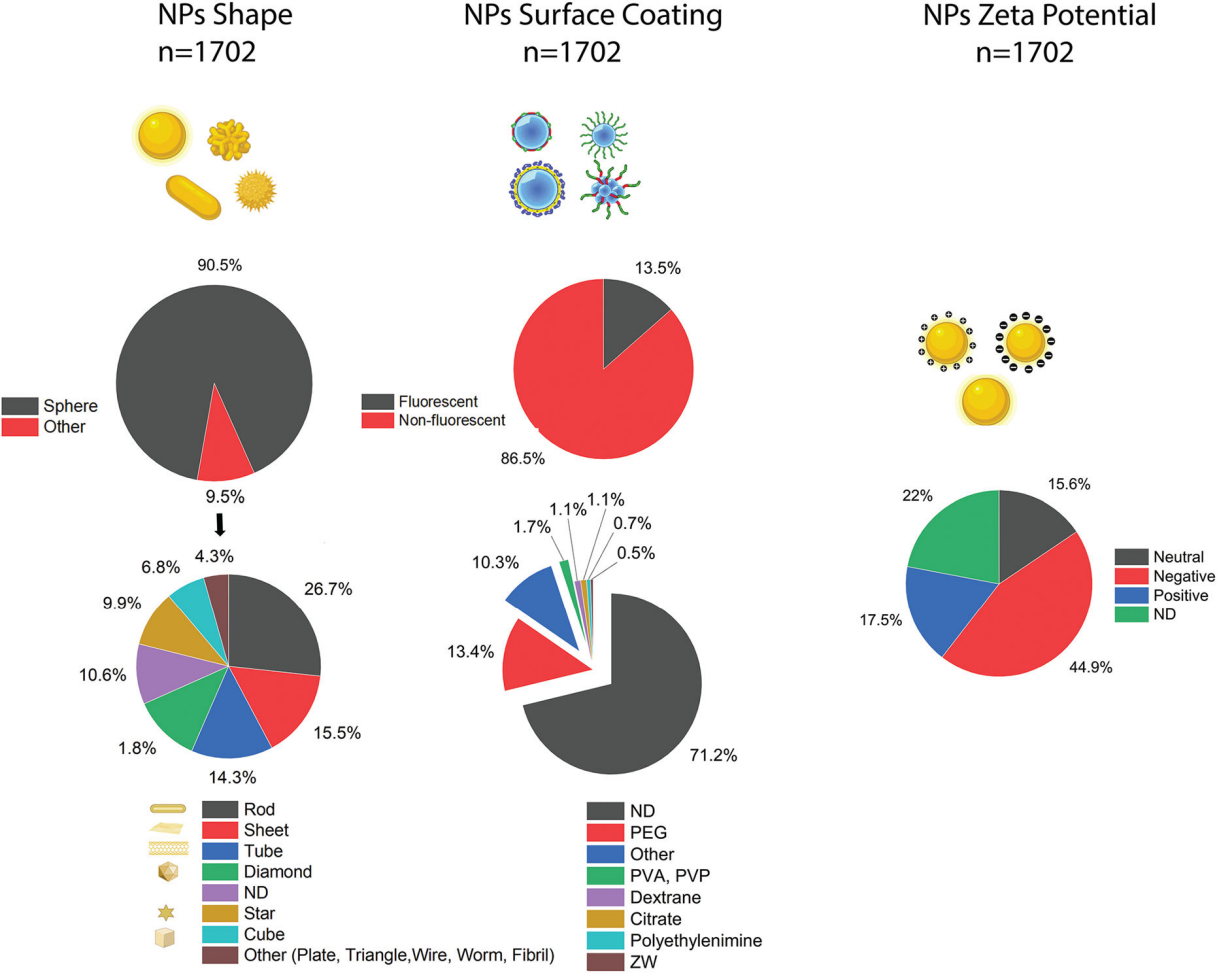
Overview of three major characteristics of NPs—shape, surface coating, and charge—used in nanomedicine studies that directly or indirectly discuss the formation and evolution of protein corona on NPs; ND: non-disclosed.

Of the protein corona literature we surveyed, most NPs (>90%) were spherical, though other shapes such as rod, sheet, and tube have also been investigated. The majority of NPs (71.2%) had no surface coatings. (Figure 3). A broad range of NPs with different zeta potentials have been investigated, therefore we created two categories of NPs based on charge: those semi-neutrally charged with zeta potential between −10 and +10 mV (15.6%), while the significant part (45%) was negative. Over 22% of the NPs used in these studies (376 out of 1702) had no reported zeta value. Detailed information on other analytical characterization techniques employed is presented in Table S1, Supporting Information.

**Figure 4.**
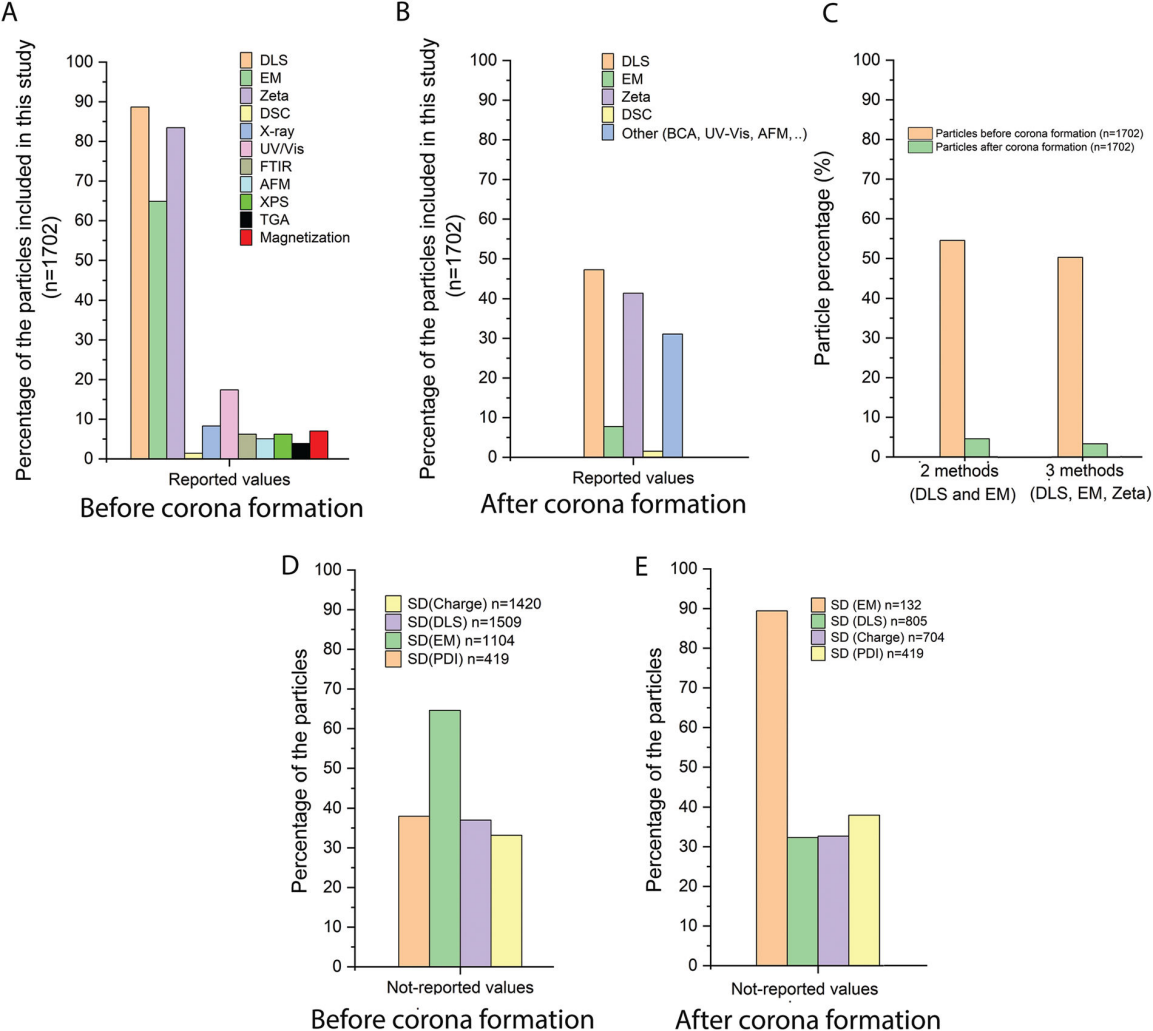
Overview of characterization of NPs before and after corona formation. An overview of the characterization tests done on nanoparticles A) before and after B) formation of protein corona in the 470 manuscripts included in this study. DLS, EM, and zeta potential are the major characterization methods performed on NPs. Other characterization tests relate to the chemical properties or functional properties of the nanoparticles and are not performed for most nanoparticles. C) The use of at minimum two complementary characterization methods was tested for the parameter of NP size. While almost half of reported studies (54.5%) used the recommended two methods for NP size determination, fewer than 5% of nanoparticles following protein corona formation has been characterized by two complementary size analysis methods. D) SD as an indicator of repeatability of the experiment is not commonly reported in NP characterization experiments. Around 40% of manuscripts (including 1702 NPs) did not report SD for key analyses such as DLS, zeta potential, and PDI. Data from EM experiments is generally not performed according to relevant standards such as ISO 21 363: “Nanotechnologies—Measurements of particle size and shape distributions by transmission electron microscopy”. Most nanoparticles (64.5%) had no reported values for SD in EM analysis. E) The reporting of SD drops following protein corona formation on NPs, which is an indicator of poor experimental repeatability of the nanoparticle protein corona literature and confirming that coronated nanoparticles are under characterized.

We then looked at the physicochemical properties of the NP protein corona studies in the literature. Figure 4A shows the various analytical and characterization tests performed on the NPs used. The major analytical tests related to assessing NP size, morphology, and charge are dynamic light scattering (DLS), electron microscopy (EM), and zeta potential studies. DLS is the major reported characterization technique for size, with <88% of NPs having reported DLS data. NPs’ charge is the second most frequent analytical technique (83% of NPs, or 1420 out of 1702). EM is the third most frequent analytical methodology for size measurements, with 64% of the NPs reporting size values as determined by EM. Data on chemical and other characterization techniques is scarce even though 82% of the NPs are synthetic (Table S1, Supporting Information). Although some characterization techniques such as magnetization are specific to NPs with magnetic properties, analytical techniques such as Fourier-transform infrared spectroscopy (FTIR), ultraviolet-visible spectroscopy (UV/vis), thermogravimetric analysis (TGA), or X-ray photoelectron spectroscopy (XPS) are not material specific and can be used for a diverse range of NPs.

We also analyzed whether the literature reports surveyed performed analytical characterization of the NPs after protein corona formation (Figure 4B). We find that DLS and zeta potential are the major techniques used for characterization of the coronated NPs, yet fewer than 50% of NPs have these values reported. The use of EM for analysis of coronated NPs is not common; fewer than 10% of coronated NPs have reported size values using EM.

Due to their complexity, the characterization of nanomaterials is not easy, and often two or more analytical methods need to be employed. For example, in line with other regulatory agencies such as the European Food Safety Authority (EFSA) and the Scientific Committee on Consumer Safety (SCCS), the Scientific Committee on Emerging and Newly Identified Health Risks (SCENIHR) also recommends at least two distinct characterization methods of size determination, one being EM, are recommended to determine the size of nanomaterials. Hence, we also calculated the percentage of NPs for which there are reported values for two or more characterization techniques for size (Figure 4C). Our results revealed that 54% of the NPs in these studies reported size using both DLS and EM. Similarly, only 50% of the NPs had reported values for DLS, EM and zeta potential. The characterization of coronated NPs even less complete, with only 4.5% of NPs having reported values using both DLS and EM (Figure 4C).

The standard deviation (SD) of a series of experimental results is a measure of repeatability. Thus, we also analyzed whether SD was reported for critical parameters such as NP size and charge (Figure 4D). More than 38% of the NPs had no reported SD for polydispersity index (PDI). Similarly, 37% and 33% of the NPs had no reported SD for DLS and zeta potential measurements, respectively. For EM, 65% of the NPs has no reported SD.

The reproducibility of data acquisition for coronated NPs is also difficult to assess, as about one third of NPs do not report SD for DLS or charge measurements. Almost 90% of EM studies on coronated NPs include no SD values (Figure 4E). All these findings indicate that the protein corona literature is inconsistent in its approach to characterizing both the NP and the coronated NPs, in a manner that likely compromises the reliability and reproducibility of protein corona composition reports.

**Figure 5.**
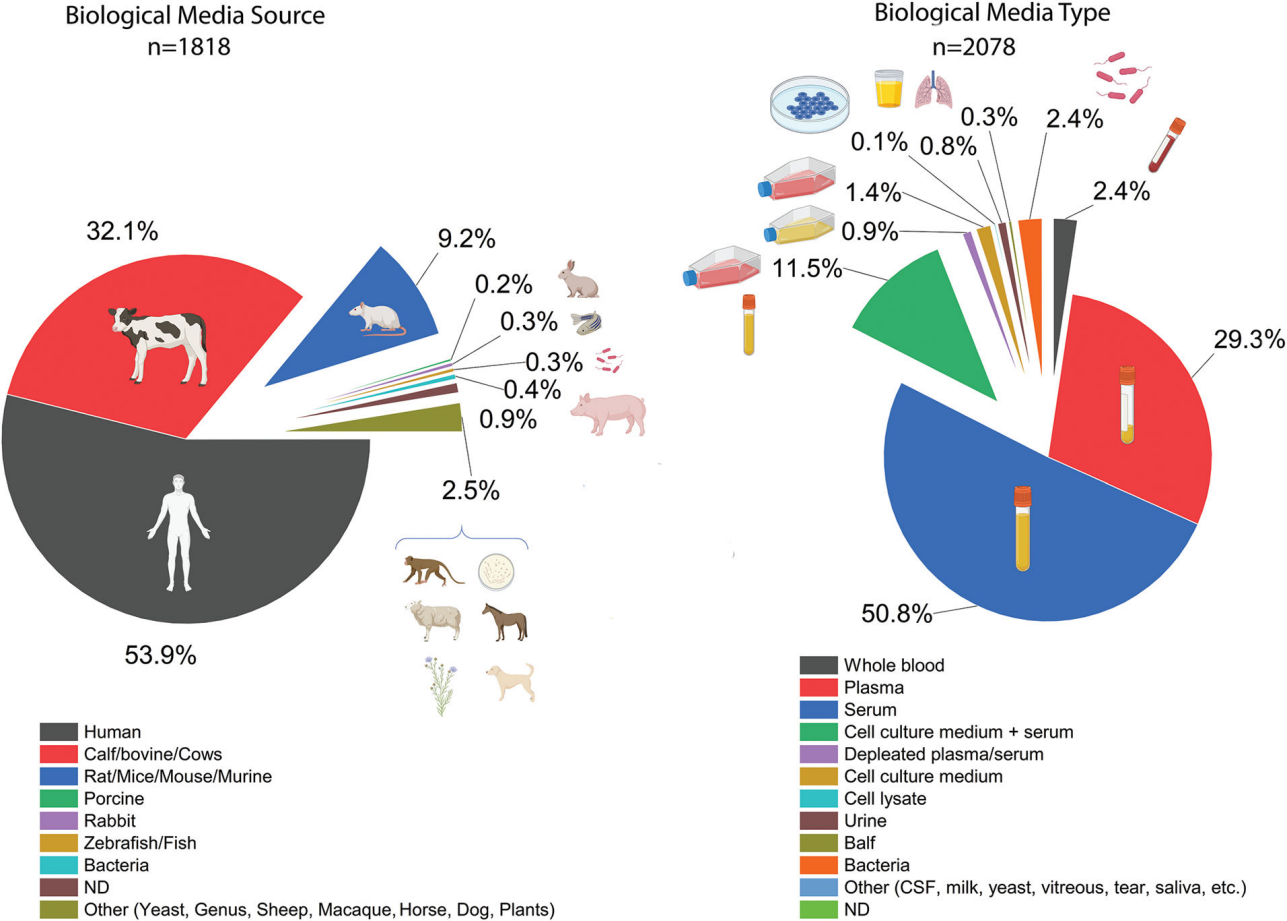
Overview of biological media sources used in obtaining and studying protein corona formation; ND: non-disclosed.

One of the critical parameters that significantly affect the robustness and outcomes of protein corona analysis is the source and type of the protein sources employed (Figure 5).^[4]^ For the 470 papers involved in this study (see Figure 1 for details), a total of 1826 biological media sources were identified, with human (53.7%) and bovine-derived biological materials (32%) forming the vast majority, followed by murine-derived materials (9.5%). Serum (51.4%) and plasma (29%) were the major biological materials used, followed by cell culture medium (11.4%). Urine, bronchoalveolar lavage fluid (BALF), CSF fluid, milk, tears, saliva, depleted plasma or serum, bacteria, or cell lysate comprised the remaining 8.17%.

Through our analysis of the employed biofluids for protein corona studies, we noticed that fewer than 1% of studies included essential information on biofluid characteristics. Examples of omitted information for plasma biofluids include type of blood extraction devices used, handling, storage, and the sex, age, and health spectrum of blood donors.^[26-33]^ This is important information to be missing from the protein corona literature, as studies reveal that even subtle differences in the type and composition of biological fluids (which can stem from any of the factors listed) can significantly change the protein composition formed on the nanoparticle corona.^[3,4,26]^

**Figure 6.**
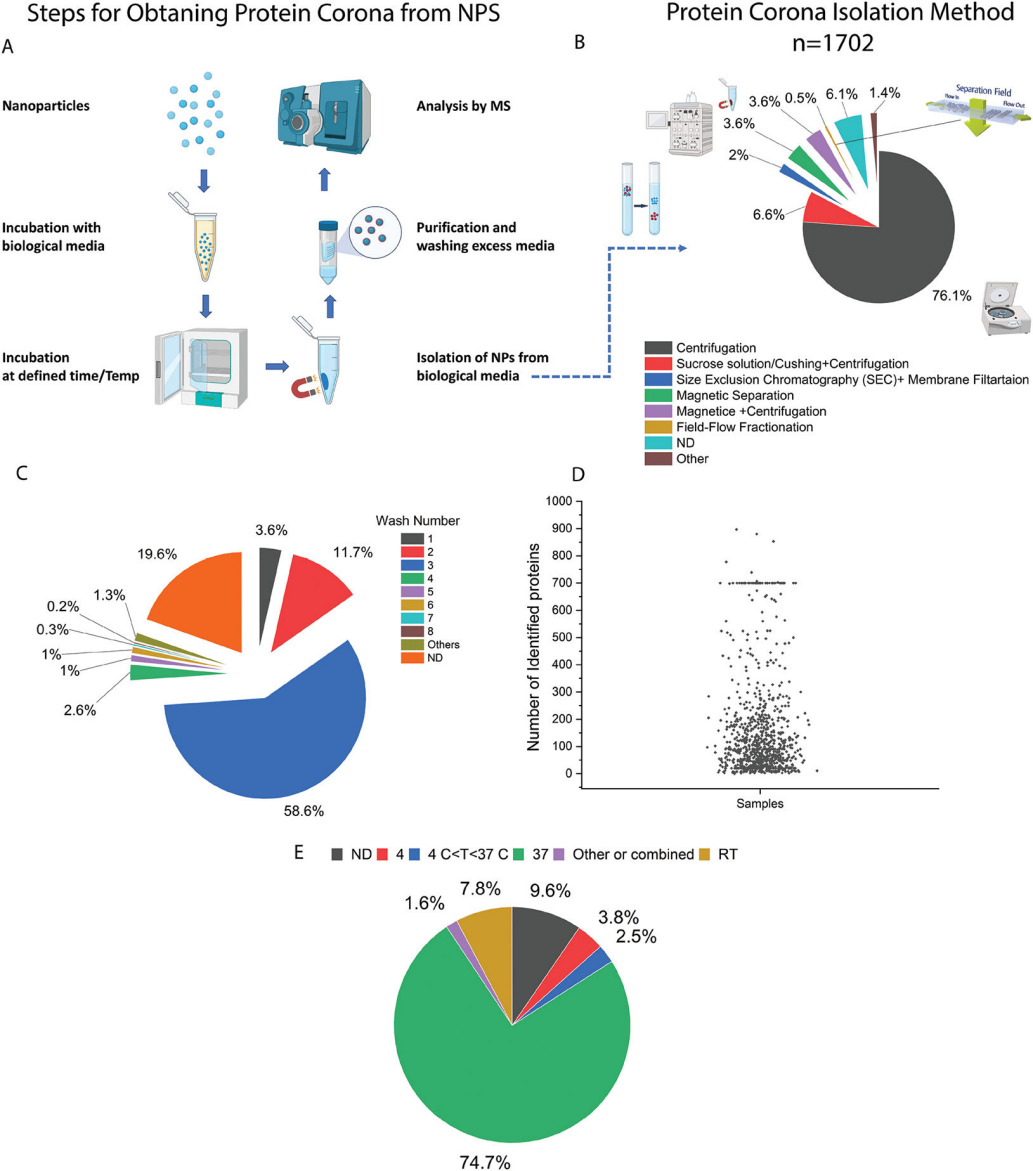
A) Summary of a general experimental workflow for obtaining/collecting the protein corona from NPs. 1) Incubation of NPs with biological media for a pre-defined amount of time and temperature, 2) isolation of coronated nanoparticles, 3) purification to remove excess media and unadsorbed proteins, and 4) characterization of protein corona by an analytical method such as LC-MS. B) Analytical methods used for obtaining biomolecular corona from NPs (*N* = 470 manuscripts). The methods are classified into six major methods consisting of centrifugation-based, gradient centrifugation, size exclusion chromatography, magnetic separation, combined methods (magnetic and centrifugation), and field flow fractionation. Simple centrifugation is the prevalent analytical method used for protein corona analysis. After centrifugation, gradient centrifugation followed by magnetic separation is also other common method for the isolation of NPs from biological media. C) The wash number used in the studies with the majority of nanoparticles being washed three times. A non-negligible proportion of studies did not report wash number (19.6%) or reported fewer than three washes (3.6 and 11.7 % for 1 and 2 washes, respectively). D) The number of proteins identified in the protein corona, including 938 nanoparticles in this analysis. For those nanoparticles which have several values reported in Table S1, Supporting Information, the largest number has been selected and included in the graph. E) Reported temperature for protein corona incubation (*n* = 1702 nanoparticles); ND: non-disclosed.

It is increasingly accepted that poor methodology in collection of coronated NPs can significantly increase the risk of errors, data misinterpretation, and a lack of reproducibility.^[4,13,14]^ The general experimental workflow to measure the NP protein corona is outlined in Figure 6A. We analyzed 470 papers with regards to the methodology used for collection of protein corona–coated NPs. The outcomes showed five main methods: centrifugation-based, density gradient centrifugation, size exclusion chromatography, magnetic separation, and field flow fractionation. Centrifugation was the most widely used for 1702 NPs (76%) and also for the 200 lipid-based NPs (80%) (Figure 6B).

However, the use of centrifugation for protein corona separation may have some limitations. For example, due to their buoyancy, pelleting soft NPs such as liposomes and lipid NPs alone is challenging using table-top centrifugation speeds. However, in some cases the formation of the protein corona can increase their density and improve collection by centrifugation. The centrifugation rate, however, needs to be carefully optimized to avoid structural damage of soft NPs and/or excess protein sedimentation that leads to experimentally-biased protein corona datasets. For example, density gradient ultracentrifugation can have high levels of protein contamination from non-adsorbed proteins, as proteins of similar density such as lipoproteins may also separate to the same layer as the NPs, and may not be recovered and thus missed in analytical quantification.^[12]^ As such, the centrifugation method, in general, makes corona-coated NPs prone to protein impurities. For example, using a combination of cryo-electron microscopy, cryo-electron tomography, and image simulation, it was recently discovered that the protein corona layer on polystyrene NPs after centrifugation may contain a significant amount of small, agglomerated impurities (≲10 nm) unassociated with the corona composition.^[34]^

**Figure 7.**
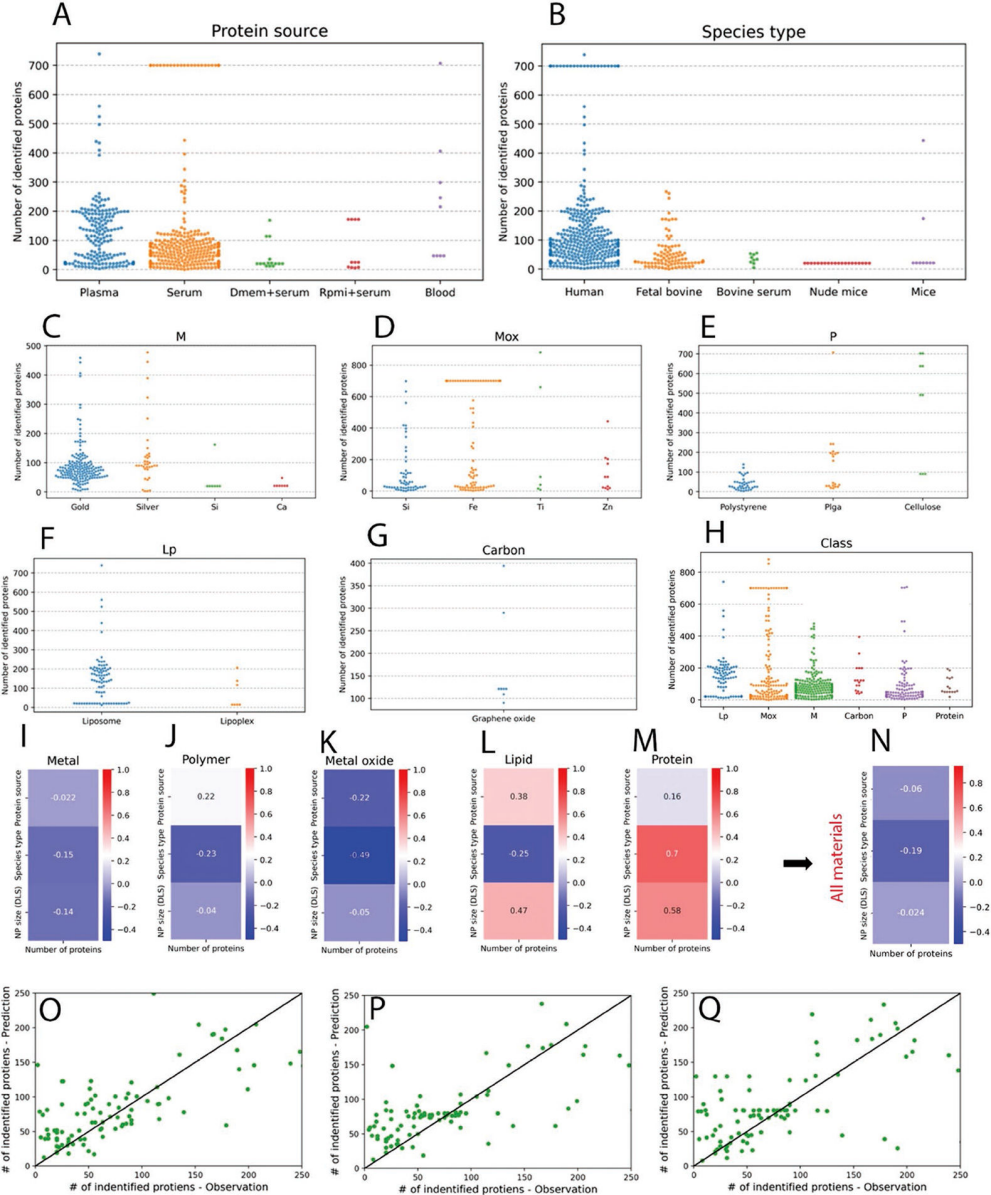
Analysis of the correlation of number of identified proteins (NIP) with experimental parameter in protein corona studies. A,B) NIP using studied NPs depends on the source and type of medium. The majority of media used in NIP studies are either plasma or serum from human or bovine sources. C–G) the correlation of NIP in each sub class of studied materials, metal, metal oxide, polymer, lipid, and carbon. H: the correlation between NIP and classes of materials including all subclasses. I–M) the statistical correlation between NIP and protein source, type and particle size (DLS) for each class of material. N) the statistical correlation between NIP and protein source, type, and particle size (DLS) for all materials including subclasses. O–Q) The correlation of Inputs: NP Size (DLS), protein source, species type and Class and output of NIP using three models; Random Forest regressor 200 trees, XGBoost 200 trees and SVM RBF kernel, *C* = 1000.

An important step in protein corona purification is washing excess protein from NPs. Therefore, we evaluated how well the washing step was implemented in protein corona preparation (Figure 6 C). While most papers used 3 washes (58.6%), which is recommended for reducing impurities,^[35]^ more than a third of papers (34.9%) either did not report the number of washes or used fewer than three washes for preparation of corona coated NPs. Figure 7D shows that a large range in the number of proteins was detected for *N* = 938 NPs. Several factors could influence the number of proteins identified in the corona layer, including the type of sample preparation for proteomics analysis (in-gel digestion, in-solution digestion, filter-based methods such as filter-aided sample preparation (FASP)), the type of mass analyzer, and the resolution of the mass spectrometry instrumentation.^[36]^ Another important factor in the protein content of the corona layer is temperature.^[37]^ In this analysis we found that physiological temperature of 37°C is by far the most common temperature used for NP and biofluid incubation (74.7%). No incubation temperature was reported for around 9.6% of NPs, and the remainder used a range of temperatures (Figure 7E).

Number and identity of detected protein in protein corona composition is one main aspect of protein corona studies which determines the protein composition of the corona layer, therefore, the fate of NPs in the biological stream. Therefore, we further studied and analyzed the numbers of identified proteins per various classes of NPs and other main factors affecting the protein corona composition, that is, protein source, protein type, and particle size. Figure 7A, B shows the observation of numbers of identified proteins in relation to protein source and type. Overall, as expected, the data shows that, on average, the use of human plasma and whole blood provides the highest numbers of identified proteins in protein corona (as the plasma contains higher numbers of proteins compared to the other sources including serum). Most of the studies on identifying protein numbers have been conducted on human biological fluids, which is essential for better understanding of the biological identity of NPs in clinical trials. Figure 7C-H shows the observation of numbers of identified proteins for each type of used NPs, revealing that most of the studies that identified protein number in corona layer have been conducted on gold, silica, polystyrene, and liposome NPs.

We, however, need to emphasize that the numbers of identified proteins are strongly depended to several factors including the physicochemical properties of NPs, the type of protein source, incubation environment, and protocols and procedures used during liquid chromatography coupled to mass spectroscopy (LC-MS/MS). For example, it was recently demonstrated that even LC-MS/MS analysis of identical protein corona coated NPs by various proteomics centers ended up with substantial heterogenicity in the numbers of identified proteins.^[36]^ More specifically, for the identical human plasma corona coated polystyrene NPs that were analyzed by 17 distinct LC-MS/MS core facilities, 4022 unique proteins were identified while only 73 (1.8%) were shared across the core facilities.^[36]^ This shows the critical need for development of standard LC-MS/MS workflow for protein corona analysis.^[36]^ In addition, studies related to the analysis of protein corona should report the minimum reporting requirements for proteomics.^[38]^

We next attempted to analyze the statistical correlation between the number of identified proteins and protein source, type and particle size (DLS) for each class of material. Without having the details of parameters affecting the numbers of identified proteins in corona (which is one of the major shortcomings of the current literature), we used two correlation methods, that is, Pearson correlation and Regression analysis. Pearson correlation^[39]^ which measures the linear correlation between two variables is used to calculate the correlation between continuous variables, that is, NP size (DLS) and number of identified proteins (NIP). The Pearson correlation ranges from −1 to 1 with 0 showing no correlations and values close to −1 and 1 representing strong negative and positive correlations, respectively. For categorical variables, namely, protein source and species type, the Point Biserial Correlation (PBC)^[40]^ method is used. PBC (mathematically equivalent to Pearson correlation) measures the correlation between a continuous (e.g., NIP) and a binary categorical variable (e.g., Protein source and Species type.) The results of this analysis for each class of materials are shown in Figure 7I-M. Figure 7N and shows the correlation of all NPs from combined classes versus DLS, protein source and protein type. The results generally indicate that there is poor correlation between NIP and chosen variable in this study between various class of materials.

To further investigate the relationship between experimental settings and their effect on NIP, we conducted a regression analysis. Specifically, 3 input variables, NP size (DLS), protein source, and species type, and one output variable, NIP, were selected and used for training various machine learning models. Random forest,^[41]^ XGBoost^[42]^ and SVM^[43]^ regressor were chosen for this study as they often exhibit high performance in case of limited and low dimensional data and are robust against overfitting. The dataset is split into training (75%) and test (25%) sets. During training, the models only have access to the training set, while the test set is kept aside to be used for evaluating the models’ generalization performance after the training. In this study, root mean squared error (RMSE) is selected as the evaluation metric. In regard to hyperparameters, 200 trees are selected for both Random forest and XGBoost models, and RBF kernel penalty term (C) of 100 are chosen for SVM.

Once the machine learning models are trained, the test set is then used to evaluate their performance on “unseen” data. Figure 7O-Q illustrates the Pareto plot along with test RMSE for each model. The *x*-axis represents the observations (true values) while the *y*-axis depicts the model’s predictions. The closer the points to the 45° reference line, the better the performance of the model. The RMSE values range between 0.55 and 0.71. Though not very high, these values show that the models were able to draw some mappings between inputs and outputs to some extent. This can also be witnessed in correlation analysis where for various combinations of inputs and output we can observe a high degree of correlations (e.g., 0.7 PBC between Species type and NIP under the Protein sub-category.) These observations can support the idea that the reported NIP across different papers investigated in this study can be substantially affected by the experiment setting variables.

## 2. Conclusions and Future Perspectives

Robust characterization of nanomaterials and biological fluids together with accurate methodological approaches to study the formation and subsequent analysis of the protein corona are essential steps in achieving a robust literature database towards designing NP-based therapeutics with higher translatability into clinical practice. Our comprehensive overview of the current protein corona literature on various types of NPs reveals the need for conducting studies with more robustness NP characterization, improving experimental repeats, and using methodologies that minimizes protein contamination and nanoparticle aggregation during the protein corona formation and collection processes. The NP corona formation in each class of nanomaterials has utmost importance specially if there is a potential of translation into a product. For example, amongst all class of NPs, lipid based NP are the most commonly utilized NPs in the clinic, yet their soft core structure often presents a hurdle for NP analysis. Hence, more reliable and accurate protein corona information for lipid-based NPs may enable scientists to develop safer and more efficient lipid-based nanoformulations. In particular, detailed information on the protein corona decoration at the surface of lipid-based NPs may improve our understanding of liver accumulation of these NPs^[44]^ and could generally enable better design-based approaches to their bio-distribution, biocompatibility, and payload delivery outcomes in vivo. In another example, compositional variations in lipid-based NPs were shown to significantly change their targeting efficacies to specific organs.^[45,46]^ The complete replacement of a conventional helper lipid with an alternative anionic or cationic lipid was shown to cause a pronounced and consistent shift of lipid NPs biodistribution in vivo from to either the mouse spleen or lung.^[47]^ The proposed mechanism for such a striking shift in NP fate in vivo is that NPs formulated with neutral or anionic helper lipids can be uptaken by epithelial and immune cells to the spleen or lung, respectively. Robust information on the protein corona profiles of these NPs are essential to define the exact mechanism of action for these and other similar NPs.^[48]^ A recent example in which protein corona information was used to design NP therapeutic outcomes was in the use of N-series lipid-based NPs (containing an amide bond in the tail), which were capable of selectively delivering mRNA to the mouse lung, in contrast to the previous discovery that O-series lipid-based-NPs (containing an ester bond in the tail) deliver mRNA to the liver.^[49]^ By analyzing the protein coronas formed on the liver- and lung-targeted lipid-NPs, this study showed that a group of unique plasma proteins specifically absorbed onto the surface of each NP type which contributed to their biodistribution outcomes.^[49]^

These studies exemplify the importance of protein corona compositional information for guiding NP therapeutic design. However, NP protein corona datasets currently exhibit too much variability to be used for ab initio design of NP outcomes in vivo. Our analysis herein shows that for lipid-based protein corona analyses, the use of well-validated protein sources, and designing protein corona experiments that consider the NP therapeutic administration route is essential to building a more robust literature dataset. Lipid-based nanotherapeutics have a wide range of administration routes including intramuscular, intratumoral, intravenous, intranasal, and oral and the biofluids these NPs will be exposed to in vivo can vary greatly.^[50-56]^ As such, we anticipate the protein corona formed for lipid-based NPs administered through different routes would be unique, and it is important to develop experimental methodologies that decrease the experimental variance we have unearthed herein to enable measuring the possibly minute differences in protein corona compositions across NP types and biofluids.

Improving the robustness of characterization and methodological approaches in protein corona preparation and analysis (e.g., by using more standard characterization strategies) may lead to most accurate and reproducible data on the safety and biological fate/efficacy of nanomedicine products. In other words, by considering and standardizing robust and accurate characterization and methodological approaches for protein corona preparation and analysis,^[3,4,13,14]^ we can gain a better understanding of nano-bio interactions on the surface of clinically relevant NPs. In addition, our analysis revealed that a considerable portion of the employed protein source for protein corona studies is human plasma, which is the most relevant protein source for clinical translation of nanotechnologies. However, more detailed information on the plasma donors should be provided in future studies, as many factors including health spectrum and sex can significantly affect the composition and function of protein corona.^[26,32,57]^ Achieving such robust nano-bio information will help the scientific community design safer and more efficient therapeutic/diagnostic NPs^[4]^ and also address the thus-far inadequately explained^[58]^ biological outcomes of clinically relevant NPs.^[59]^ The nanomedicine community also needs to develop standard methods for mass spectroscopy analysis of protein coronas, as very recent findings revealed the critical role of heterogeneity in proteomics analysis of nanoparticle protein corona.^[36]^ Finally, a more robust understanding of the protein corona formed on the surface of lipid-based NPs may facilitate development of efficient, safe, and immune-cell-specific mRNA delivery systems that could pave the way for introduction and widescale use of both current and next-generation robust mRNA-based immunotherapeutics.^[59]^

## Supplementary Material

SI
